# Multi-source dataset of NO_2_ and PM from mobile and fixed sensors in Rouen (France)

**DOI:** 10.1016/j.dib.2026.113025

**Published:** 2026-06-24

**Authors:** Michel Bobbia, Jean-Michel Poggi, Bruno Portier, Emma Thulliez

**Affiliations:** a ATMO Normandie, 3 place de la pomme d’or, Rouen, 76000, France; b LMO, Université Paris-Saclay, Fac. Sciences Orsay, Bat. 307, 91400, Orsay, France; c Dept SD, IUT Paris-Rives de Seine, Université Paris Cité, Paris, France; d INSA Rouen Normandie, Normandie Univ, LMI UR 3226, F-76000, Rouen, France; e UMR MIA Paris-Saclay, AgroParisTech, Université Paris-Saclay, 91120, Palaiseau, France

**Keywords:** NO2, PM10, PM2.5, Mobile Sensing, Low-cost sensors network, Air quality, AtmoTrack, AirSensEUR

## Abstract

This article presents a dataset bringing together measurements of nitrogen dioxide (NO_2_) and particulate matter (PM_10_ and PM_2.5_), obtained from mobile and fixed devices, collected using low-cost sensors and monitoring stations in Rouen (France). These measurements were obtained as part of the TIGA research project, ‘Rouen: Smart Mobility for All’, launched by Métropole Rouen Normandie to improve its transport network and reduce its impact on air quality (this research project is still in the experimental phase). As part of this project, and to complement the research carried out to date using fixed sensors, it has been decided to incorporate mobile sensors, both to enhance spatial measurements representativeness, and to investigate how multi-hop calibrations can be carried out using this new mobile measure network.

Therefore, the measurement network consists of ten mobile AtmoTrack sensors installed on buses (measurements every 10 s), eighteen AirSensEUR sensors deployed on traffic lights (measurements every minute), and six fixed monitoring stations (measurements every 15 min). It also includes measurements from two AtmoTrack sensors (measurements every 5 min) installed in the vicinity of a reference station. All data were collected over a period of more than ten months, from January 15, 2025, to November 30, 2025.

The deployment of these ten mobile AtmoTrack sensors followed a two-month feasibility study (October 2023) aimed at assessing the added value of a mobile sensor network for PM_10_ measurements [1].

In addition to NO_2_ and PM concentrations, the dataset includes, for mobile sensors, information related to bus speed and GPS position, as well as internal meteorological parameters (relative humidity, temperature, and atmospheric pressure). For fixed sensors, in addition to these three meteorological parameters, measurements of additional atmospheric gases are available (Ox, NO, and CO).

This dataset constitutes a relevant resource for the development and evaluation of calibration methods for multisource networks, as well as for the study of heterogeneous data fusion. The availability of mobile measurements can also help public and private stakeholders better identify their needs and implement targeted actions aimed at improving air quality—particularly for NO_2_, PM_10_ and PM_2.5_—and consequently the health of urban populations.

Specifications Table

**Table:** 

Subject	Earth & Environmental Sciences
Specific subject area	Air Quality Monitoring
Type of data	Tables (.csv files) of raw measurements and coordinates.
Data collection	This dataset consists of particulate matters (PM_10_, PM_2.5_) and nitrogen dioxide (NO_2_) measurements made between January 15 and November 30, 2025, by three types of devices: - Reference sensors in 6 monitoring stations (three urban background sites and three urban traffic sites). - 10 mobile low-cost sensors, of brand AtmoTrack ([3]) located on public buses. Sensors also provide bus speed and GPS location. - 2 fixed low-cost sensors, of the same brand AtmoTrack, collocated on QDP station. - 18 AirSensEUR low-cost sensors ([2]) located on traffic lights. Sensors also provide other gases (CO, Ox, NO) measurements, temperature, relative humidity and atmospheric pressure.
Data source location	Data were collected in Rouen, Normandy, France. The coordinates (except for mobile sensors, whose coordinates are indicated in the data files) are provided in .csv files in addition to the files containing the measurements.
Data accessibility	Repository name: Mendeley Data Data identification number: 10.17632/5sphf5c592.1 Direct URL to data: https://data.mendeley.com/datasets/5sphf5c592/1
Related research article	None.

## 1 Value of the Data

• The data allows us to study the challenges associated with merging heterogeneous data from reference stations, fixed microsensors, and mobile microsensors, acquired at different time scales and over a period of almost a year (see, for example, [3] and [4]).
• This dataset can be used by the scientific community to develop, implement, and evaluate collaborative calibration methods such as multi-hop calibration applied to mobile and fixed microsensors (see, for example, [5,6] and [7]).
• The data provide a particularly relevant framework for analyzing and proposing new definitions for rendezvous between mobile sensors and fixed devices, due to the richness of the dataset and the heterogeneity of the time scales specific to the different devices (see, for example, [8] and [9]).
• Thanks to the long duration of the measurement campaign, this dataset can also be used to study the phenomenon of temporal drift in microsensors, whether mobile or fixed (see, for example, [10] and [11]).
• The availability of mobile measurements can also help public and private stakeholders better identify their needs and implement targeted actions aimed at improving air quality—particularly for NO2, PM10 and PM2.5—and consequently the health of urban populations.

## 2 Background

This dataset was compiled by Atmo Normandie, association that monitors air quality in Normandy (France). Since January 2025, ten AtmoTrack mobile sensors have been installed on buses operated by Transdev, the company that runs the public transport network in the Rouen metropolitan area. These sensors monitor fine particulate matter (PM_10_ and PM_2.5_) and nitrogen dioxide (NO_2_), thus contributing to a better understanding of air pollution in the Rouen metropolitan area. They also offer the opportunity to explore and develop new methods for calibrating sensors. This deployment follows a feasibility study conducted over a two-month period, during which two AtmoTrack mobile sensors were installed on buses. The results of this study demonstrated the value and relevance of this type of device, and the data were published [1].

The deployment of these mobile microsensors complements a network of eighteen fixed microsensors already installed on traffic lights in the city of Rouen, as part of the “Rouen Smart Mobility for All” research project initiated by Métropole Rouen Normandie.

The data presented in this article comes from measurements taken between January and November 2025. It includes measurements from all the fixed and mobile microsensors deployed, as well as those from six reference monitoring stations.

## 3 Data Description

The dataset consists of 68 .csv files of four types:

- The table 'coordinates.csv' provides the coordinates of all fixed devices (the 6 monitoring stations and 18 AirSensEUR sensors). It consists of three columns, namely 'name', 'longitude' and 'latitude', with longitude and latitude expressed in standard WGS84 system.

**Fig. 1:**
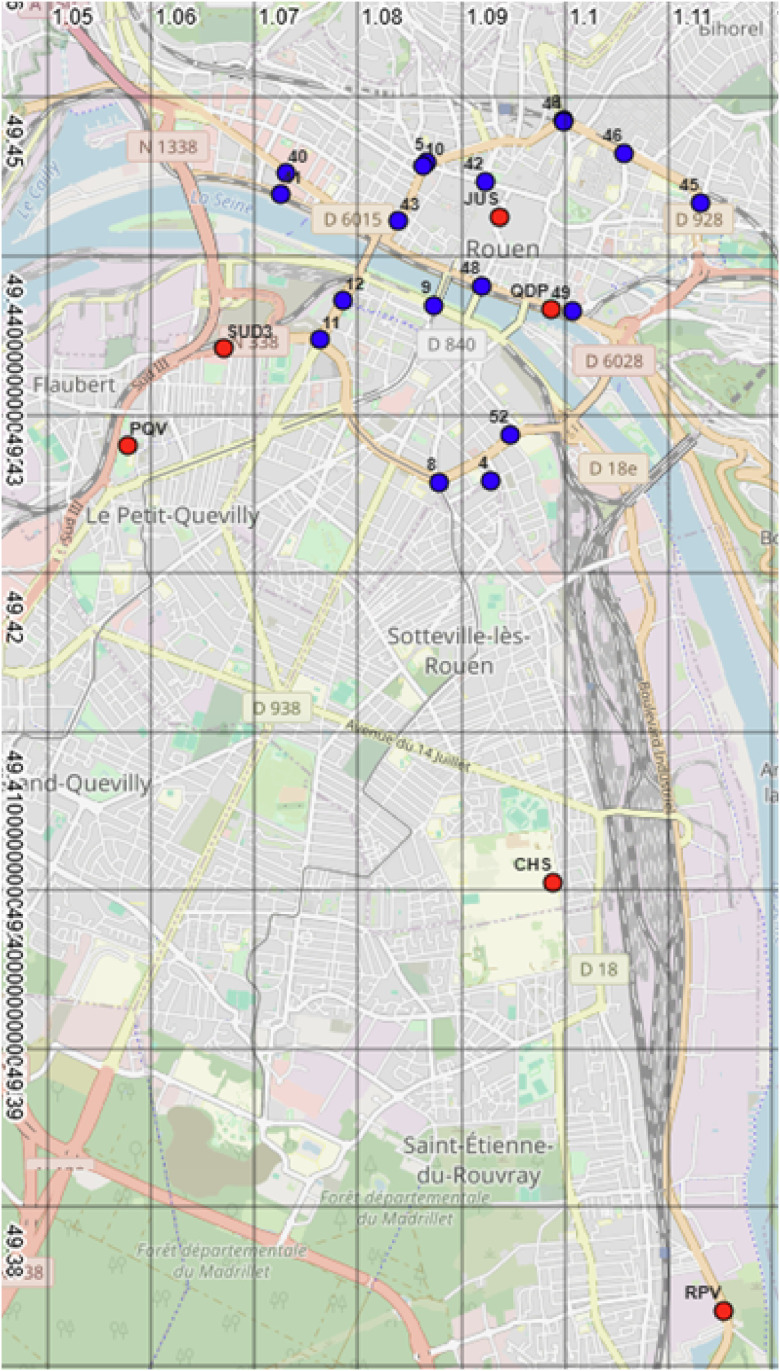
Measuring sites over Rouen city. Red points stand for fixed reference stations, while blue ones stand for AirSensEUR low-cost sensor.

Fig. 1 presents the map of Rouen, with position of each fixed device. Monitoring stations are represented by red dots and low-cost sensors by blue ones.

- The file 'reference_measures.csv' contains every measurement collected by reference monitoring stations.

The first column, 'time', indicates the moment of measurement (format UTC±00:00), then each column is named as follows 'AAA_XX' with 'AAA' the name of the station (CHS, JUS, PQV, QDP, RPV or SUD3), and XX the pollutant (NO_2_, PM_10_ or PM_2.5_). PM_2.5_ correspond to particulate matter with diameter <2.5 µm while PM_10_ have a diameter <10 µm. All measurements are given in µg/m^3^.

- Measurements from AirSensEUR sensors are stored in files named 'ASEXX_YY.csv', with 'XX' in {4:6, 8:12, 40:46, 48, 49, 52} and 'YY' being PM, GAS or MTO. We have three files per sensors leading to a total of 54 files. Each one contains the column 'date' for the time of measurement (format UTC±00:00).

PM files also contain columns 'PM10′ and 'PM2.5′ for particulate matters measurement in µg/m^3^, and validity_flag_PM10 and validity_flag_PM2.5 for the validity status code. The different validity codes are 'B' for raw data, 'N' for missing data and 'I' for invalidated data (see below).

GAS files contain the column 'date' and measurements of pollutants in columns 'NO', 'NO2′, 'Ox', 'CO', validity flag for each, as for PM. Those four parameters are measured in µV.

MTO files contains the column 'date', 'T' for temperature in °C, 'RH' for relative humidity in % and 'P' for atmospheric pressure in hPa. Here again, the validity flags are also provided for each parameter.

- Twelve .csv files, named 'ATM1.csv' to 'ATM12.csv', complete the dataset. The first ten files are dedicated to AtmoTrack mobile sensor, while the last two files are related for AtmoTrack fixed sensors located at QDP station. Files contain the time of measurement (column 'time', format UTC±00:00), the coordinates of the sensor at that time ('longitude' and 'latitude' in standard WGS84 system), and measurements of PM_2.5_ (column 'pm2.5′), PM_10_ (column 'pm10′) and NO_2_ (column 'no2′), all in µg/m^3^, for pollutant measures, and T (column 'temperature') in °C, RH (column 'humidity') in %, for meteorological measures, and 'speed' for bus speed, in km/h, for the ten mobile sensors (ATM1 to ATM10).

Additionally, the files also contain particle counts classified by size ranging from 0.3 µm to 10 µm (column 'lit0.3′, 'lit0.5′, 'lit1.0′, 'lit2.5′, 'lit5.0′ et 'lit10.0′), each column refers to a number of particles greater than the diameter indicated in his name.

All information is summarized in Table 1.

**Table 1:** Summarized content of the dataset.

Device	Variable	Unit	Timestep	File
Reference analyzer	time NO2 PM10 PM2.5	UTC±00:00 µg/m^3^ µg/m^3^ µg/m^3^	every 15 min	reference_measures.csv
Fixed sensors (AirSensEUR)	date PM10 validity_flag_PM10 PM2.5 validity_flag_PM2.5	UTC±00:00 µg/m^3^ Boolean µg/m^3^ Boolean	almost every minute	ASEXX_PM.csv *with 'XX' in {4:6, 8:12, 40:46, 48, 49, 52}*
	date NO validity_flag_NO NO2 validity_flag_NO2 Ox validity_flag_Ox CO validity_flag_CO	UTC±00:00 µV Boolean µV Boolean µV Boolean µV Boolean	almost every minute	ASEXX_GAS.csv *with 'XX' in {4:6, 8:12, 40:46, 48, 49, 52}*
	date T validity_flag_T RH validity_flag_RH P validity_flag_P	UTC±00:00 °C Boolean % Boolean hPa Boolean	almost every minute	ASEXX_MTO.csv *with 'XX' in {4:6, 8:12, 40:46, 48, 49, 52}*
Mobile sensors (AtmoTrack)	time longitude latitude pm2.5 pm10 no2 T RH speed litXX *(with 'XX' in {0.3,0.5,1,2.5,5.0,10})*	UTC±00:00 WGS84 WGS84 µg/m^3^ µg/m^3^ µg/m^3^ °C % km/h integer	almost every 10 s	ATMXX.csv *XX ranges from 1 to 12*

All the data have been examined. For the AirSensEUR data, a procedure was conducted to invalidate the following data:

- outside the range [−30; 50] for temperature;
- outside [0;100] for relative humidity;
- outside [900;1150] for atmospheric pressure;
- below percentile 1 or above percentile 99 of the raw data for gas and PM.

The raw data are kept; only the validity flag indicates the state of the data according to the above procedure. Anyone is free to apply a different procedure.

For AtmoTrack data, AtmoTrack conducted a validation procedure to invalidate NO_2_ data that is not meaningful, especially when the bus starts up after a period of shutdown and rest. We haven't carried out any further validation nor data preprocessing or filtering.

For reference data, Atmo Normandie performs the validation procedure in accordance with its usual rules. These rules are complex and governed by French regulation and national standards, as well as by the recommendations of analyzer manufacturers and the Central Laboratory for Air Quality Monitoring (LCSQA) – see for example the French standard NF EN 14,211. Highly qualified staff carry out regular maintenance on the equipment on site. In addition, a team of experts regularly validates the reference measurements at least twice a day. This validation process cannot be replicated here.

Known measurement errors for each sensor component are given in Table 2.

**Table 2:** Measurement errors provided by the manufacturers.

Device	Variable	Sensor	Measurement error
Fixed sensors (AirSensEUR)	PM10 PM2.5	OPC—N3 OPC-N3	Max coincidence probability: %concentration at 10^6^ particles/L = 0.84 %concentration at 500 particles/L = 0.24
	NO NO2 Ox CO	NO_B4 NO2-B43F OX-A431 CO-BF	500 to 850 nA/ppm in 2 ppm NO −200 to −650 nA/ppm at 2 ppm NO2 –200 to –650 nA / ppm at 1 ppm 80–130 nA / ppm
	T RH P	SHT31TE / SHT31TI SHT31HE / SHT31HI BMP280	± 0.2 °C ± 2% ± 1 hPa
Mobile sensors (AtmoTrack)	PM10 PM2.5 NO2 T RH	No information has been provided by the manufacturer.	±10% at 100–500 μg/m^3^, ±10 μg at 0–100 μg/m^3^ ±10% at 100–500 μg/m^3^, ±10 μg at 0–100 μg/m^3^ 10 ppb ou 19 μg/m^3^ ±0.5 °C at 25 °C, ±1 °C else ±3%RH
Reference Sensors (Atmo Normandie)	NO2 PM10 PM2.5	HORIBA (model APNA 370 for JUS, PQV, RPV, QDP and SUD3) or ENVEA (model AC32e for CHS) ambient NOx analyzers, and TEI (model TEOM-FDMS 1405 for RPV and SUD3), MetOne (model BAM 1020 for QDP) or PALAS (model FIDAS 200 for CHS, JUS and PQV) for PM analyzers.	The measurement uncertainty complies with the requirements of the European directives, which stipulate that it must be <15% for gases and 25% for particulate matter. The uncertainty percentages are given for individual measurements, averaged over the period considered for the limit value, with a 95% confidence level. The uncertainty applies within the range of the relevant limit value (or the target value in the case of ozone).

**Fig. 2:**
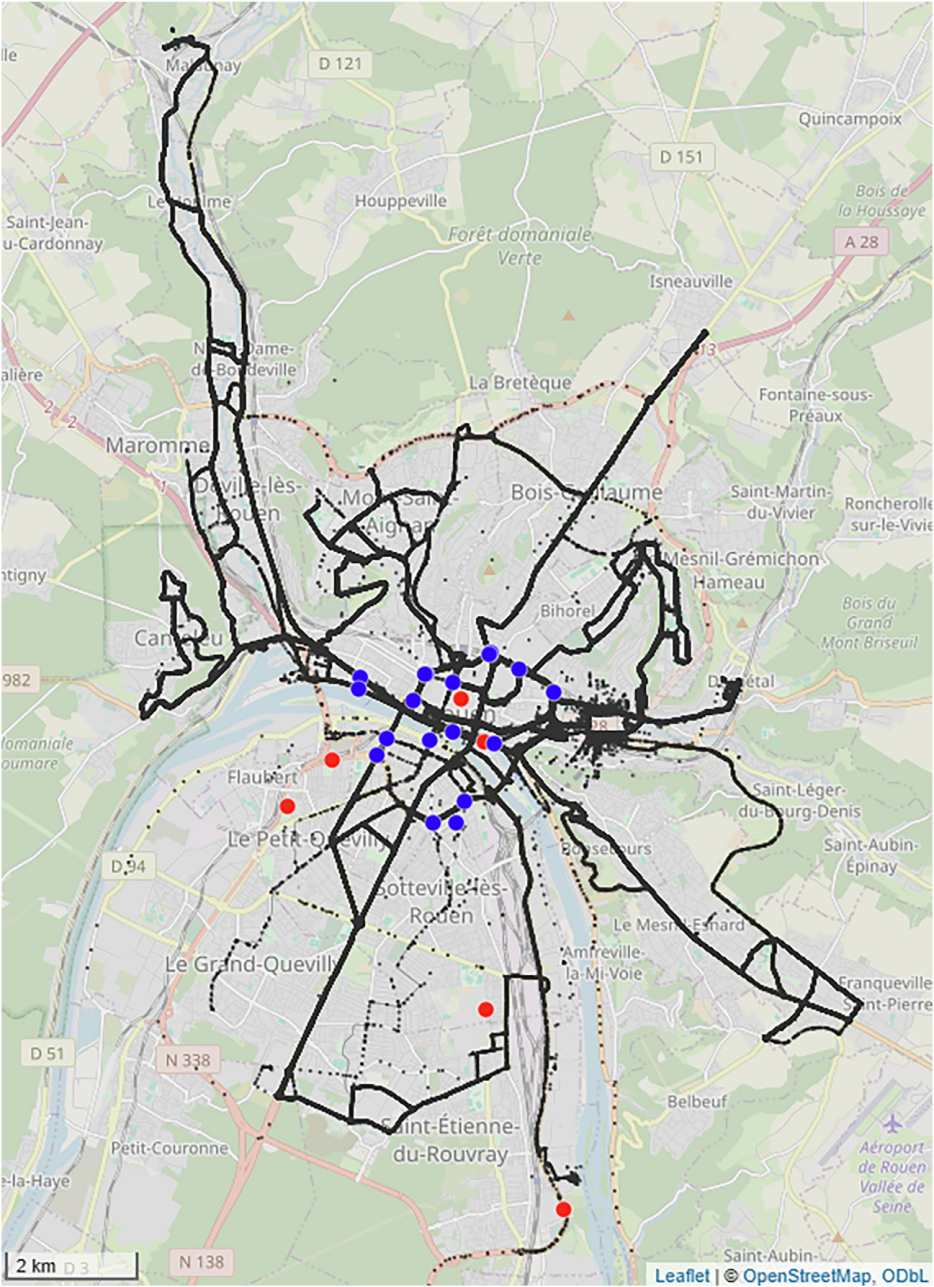
All routes followed by the ten buses between 15 January and 30 November 2025. Locations of mobile sensors at the time of measurement are represented by black dots. The locations of the fixed measurement sites are represented by red dots (reference analyzers) and blue dots (fixed low-cost sensors).

Fig. 2 shows all the routes traveled by the 10 buses during the period from January 15 to November 30, 2025. Each trajectory is made up of the location of the bus every 10 s. It can be seen that the area covered by the 10 buses covers almost the entire Rouen metropolitan area. Several buses pass near the QDP station, and one near the RPV station. It can be noticed that bus trajectories meet almost every sensor during the period, with exception of sensors ASE9 and ASE41.

## 4 Experimental Design, Materials and Methods

The monitoring network described in this article is based on the following devices:

### 4.1 Regulatory monitoring stations

Six monitoring stations, named CHS, JUS, PQV, QDP, RPV, and SUD3, managed by Atmo Normandie, are situated in Rouen. The QDP, RPV, and SUD3 stations are located at sites strongly influenced by road traffic, while the urban background stations CHS, JUS, and PQV are installed at sites representative of background pollution in the conurbation.

### 4.2 Low-cost AirSensEUR sensors

Eighteen low-cost AirSensEUR brand sensors were deployed across the Rouen area. Eight of them were initially installed in the immediate vicinity of traffic monitoring stations to allow a collocation phase. The remaining sensors were installed during a second phase, without a prior collocation period.

These sensors measure something strongly related to gas concentrations with electrochemical cells of brand Alphasense, providing an electrical signal in µV for NO_2_, NO, Ox and CO.

The meteorological parameters are made with Sensirion sensors for T (in °C) and RH (in %), and Bosch Sensortec for P (in hPa, that is to say mbar).

### 4.3 Mobile AtmoTrack sensors

Ten AtmoTrack brand sensors were installed on the roofs of ten buses in the public transport network, and two sensors of the same brand were installed at the QDP station.

The measurements obtained are converted into µg/m^3^ using a conversion algorithm developed by AtmoTrack. Sensors also provide bus speed and non-corrected GPS location. Data are transmitted every 10 s to the AtmoTrack database and are retrieved online by Atmo Normandie.

For the two AtmoTrack sensors installed at the QDP station, measurements are retrieved every five minutes.

Thanks to the richness of this dataset, numerous experiments can be conducted. For example, the AtmoTrack ATM11 and ATM12 sensors, based on the same technology and collocated at the QDP station, offer an ideal configuration for comparing their measurements and studying the repeatability of the sensors. Furthermore, the availability of reference measurements makes it possible to evaluate the performance of these sensors and develop appropriate correction or calibration strategies.

**Fig. 3:**
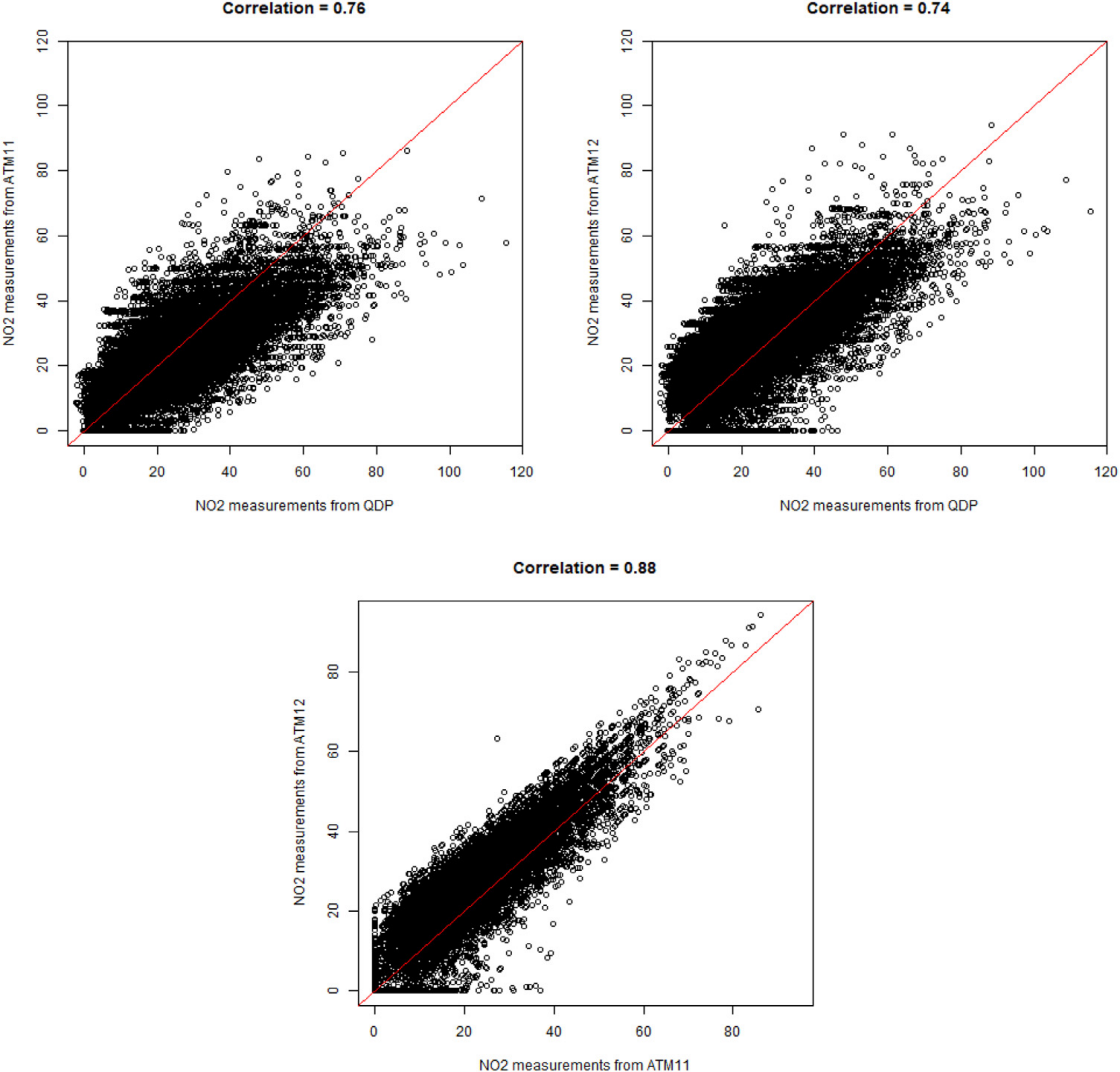
Scatter plot of quarter-hourly NO_2_ measurements at QDP reference station.

Although the ATM11 and ATM12 sensors are based on the same technology, measurement discrepancies can nevertheless be observed, justifying the interest in such analyses. Fig. 3 shows scatter plots of NO₂ concentrations provided by the three measuring devices. Measurements from the ATM11 and ATM12 sensors were aggregated at 15-minutes intervals to enable direct comparison with reference measurements.

As illustrated in Fig. 2, several buses operate near the QDP station and are likely to cross paths during their journeys. This configuration allows multi-hop calibration strategies to be considered and their performance to be evaluated.

**Fig. 4:**
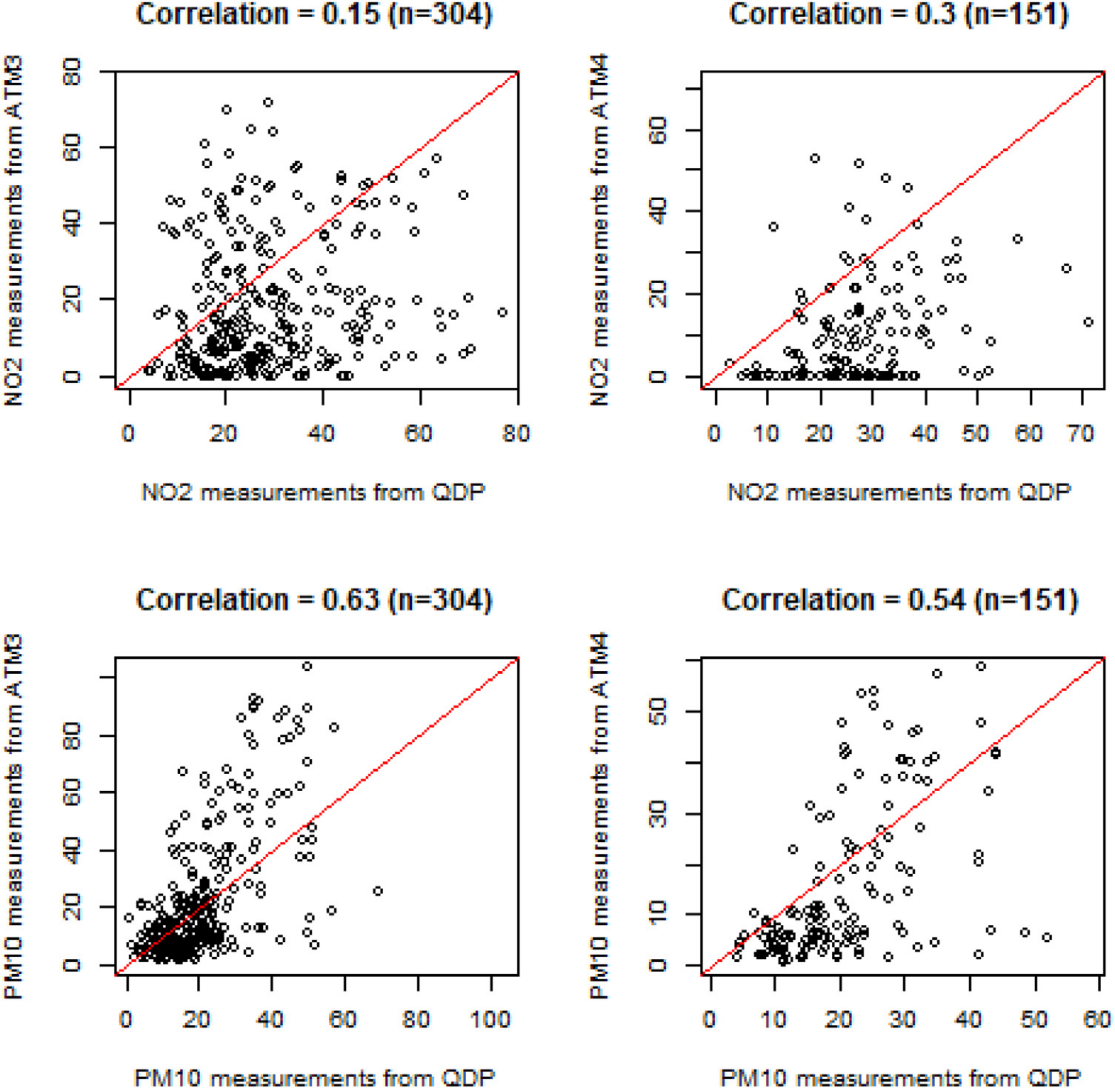
Scatter plot of NO₂ measurements for two buses each meeting with the QDP station. On the x-axis are the quarter-hourly reference measurements at QDP. On the y-axis are the measurements from the AtmoTrack ATM3 or ATM4.

Fig. 4 shows the scatter plots of the concentrations (NO_2_ and PM_10_) measured by buses ATM3 and ATM4 versus the reference measures at QDP station. The associations between measurements were made using a spatiotemporal approach defining a rendezvous. Roughly speaking, a rendezvous between two sensors is determined by their presence in approximatively the same place at approximatively the same time.

The measurements from the mobile sensors were first synchronized temporally at 5-second intervals to facilitate their temporal pairing. Indeed, although the AtmoTrack mobile sensors acquire measurements at a frequency of approximately ten seconds, the timestamps of two separate sensors are not necessarily synchronized. Each mobile measurement was then associated with the corresponding reference measurement, calculated over the quarter hour immediately preceding the mobile measurement. The spatial association of measurements was carried out by retaining only those observations for which the distance between the measuring devices was <30 m

This spatiotemporal approach can also be applied to compare measurements from mobile sensors mounted on buses that cross paths. For example, the process of establishing rendezvous between two AtmoTrack mobile sensors can be:

— Firstly, the measurements from the AtmoTrack sensors can be temporally aligned to a resolution of five seconds, assigning each measurement to the nearest date.
— Secondly, rendezvous between AtmoTrack mobile sensors can be identified by retaining only the 5 s instances where the distance between sensors is <50 m

**Fig. 5:**
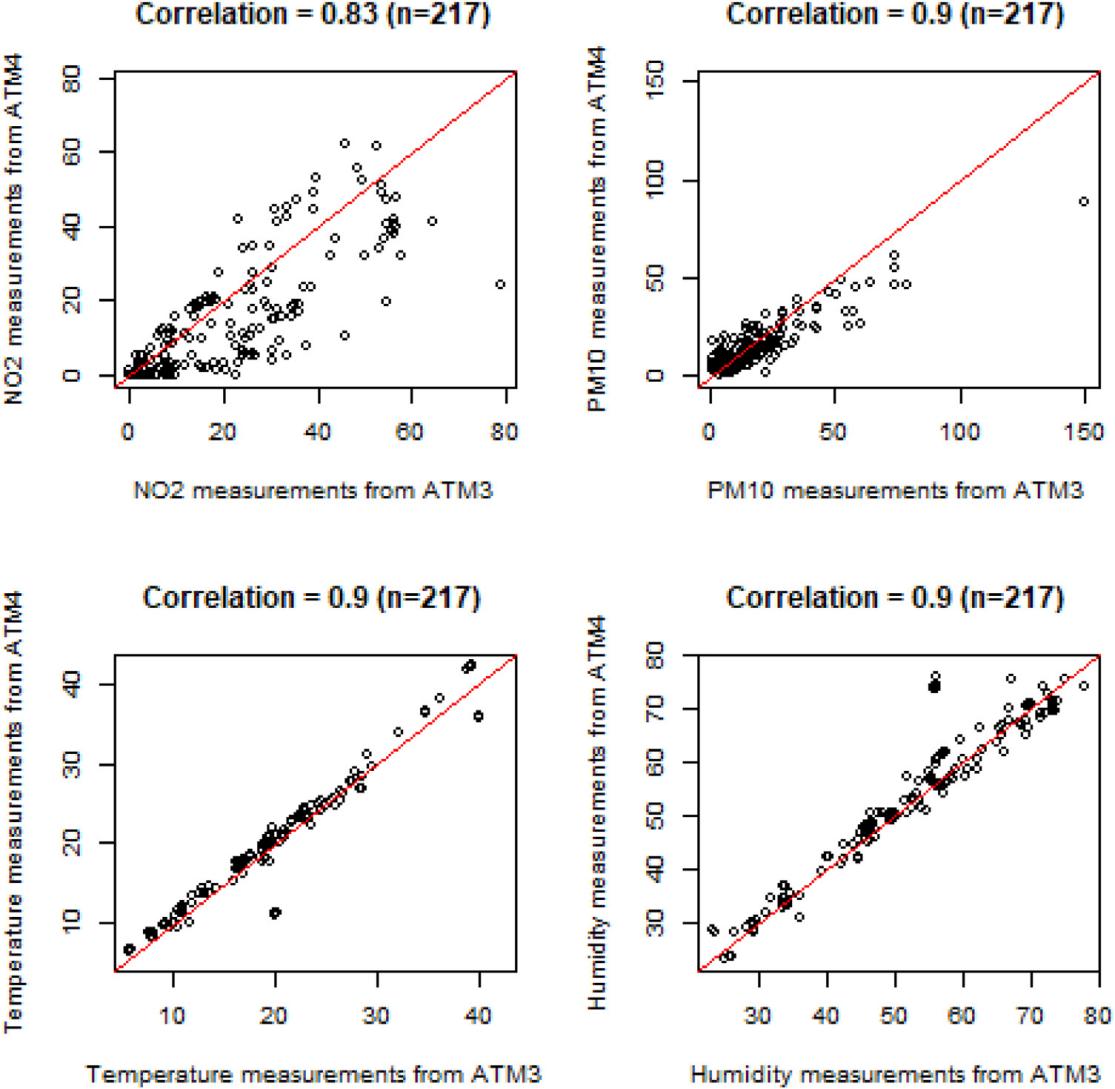
Scatter plot of ATM4 measurements against ATM3 measurements for rendezvous of <30 m

Fig. 5 shows the measurements of PM10, NO2, temperature, and relative humidity from the mobile sensors ATM3 and ATM4 when they cross paths.

**Fig. 6:**
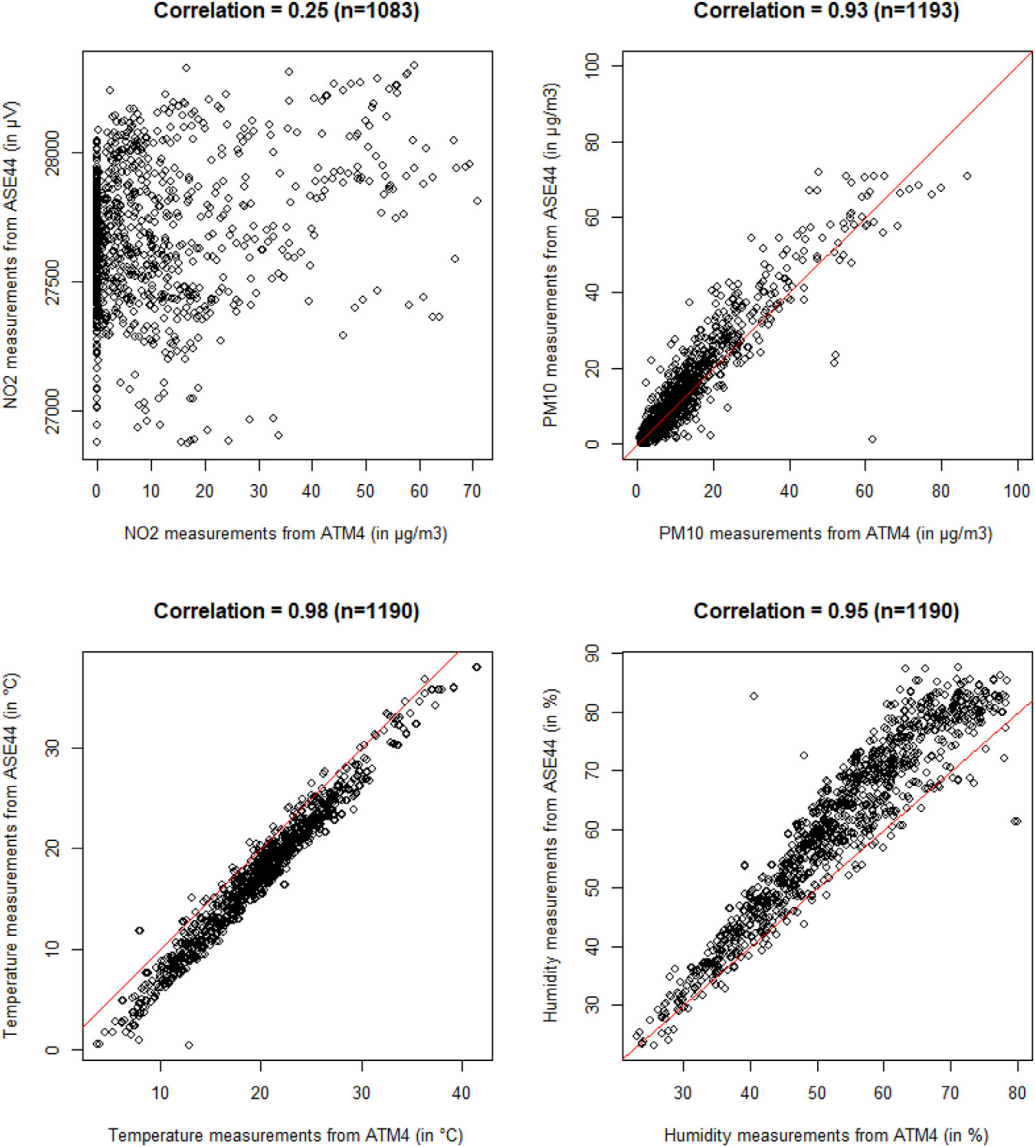
Scatter plot of ASE44 measurements against ATM4 measurements for rendezvous of <30 m

The same approach can be used to compare measurements from a mobile sensor, installed on a bus, with those from a fixed sensor installed on a traffic light, when the bus passes nearby. In this last case, measurements from mobile and fixed sensors were temporally synchronized at 1-minute intervals to facilitate their pairing. Fig. 6 shows the measurements of PM10, NO2, temperature, and relative humidity from the fixed sensor ASE44 and the mobile sensor ATM4 when the latter passes nearby. In this context, the NO2 measurements are not comparable since the ASE44 sensor provides measurements in microvolts, whereas the ATM4 sensor provides measurements in micrograms per cubic meter. This highlights the need to calibrate the fixed NO2 micro-sensor, for example, using a multi-hop calibration strategy.

## Limitations

GPS coordinates of mobile sensors have not been posteriori validated. They might show some uncorrected positioning errors.

Note: the latest data available for ATM11 dates from 2025–11–20 06:47:31; the sensor stopped working after this date and until the end of the campaign provided here, i.e. 2025–11–30.

Note also that the data for 27 June for the QDP station is anomalous, probably due to a power cut affecting the entire station; this therefore also affects ATM 11 and 12. We recommend invalidating the entire day's data.

## Ethics statement

The authors have read and follow the ethical requirements for publication in Data in Brief and confirm that the current work does not involve human subjects, animal experiments, or any data collected from social media platforms.

## CRediT Author Statement

**Michel Bobbia**: Resources, Data Curation, Writing-Original Draft. **Jean-Michel Poggi**: Writing - Review & Editing. **Bruno Portier**: Conceptualization, Supervision, Writing-Original Draft. **Emma Thulliez**: Writing - Review & Editing.

## Data Availability

Mendeley DataMulti-source dataset of NO2 and PM from mobile and fixed sensors in Rouen (France) (Original data)
